# COVID-19 Fears May Be Worse Than the Virus: A Case of Cardiogenic Shock Secondary to Post-Myocardial Infarction Ventricular Septum Rupture

**DOI:** 10.7759/cureus.8809

**Published:** 2020-06-24

**Authors:** Salem Gaballa, Avan AlJaf, Kashyap Patel, Jane Lindsay, Kyaw M Hlaing

**Affiliations:** 1 Internal Medicine, LewisGale Medical Center, Salem, USA

**Keywords:** covid-19, post mi ventricular septum rupture, left to right shunt, cardiogenic shock

## Abstract

Since the beginning of the coronavirus disease 2019 (COVID-19) pandemic, there has been a growing and justifiable fear of catching the virus from the emergency rooms, thus decreasing the hospital visits. With Virginia State slowly reopening and HCA local hospitals resuming elective procedures, the number of emergency room visits, are recovering and increasing. We report a sad and unfortunate case of an 87-year-old female who was experiencing pressure-like chest pain but presented to the emergency room five days later out of fear of catching COVID-19 from the hospital. On presentation to the ED, she was found to have an non-ST-elevation myocardial infarction, which required urgent stenting of the left anterior descending artery. Unfortunately, several hours later, she developed fatal cardiogenic shock due to ventricular septal rupture. We are reporting this case to highlight one of the many potential bad outcomes as a result of a delay in seeking necessary medical attention due to the fear of contracting the virus.

## Introduction

Ventricular septal rupture (VSR) is an uncommon but fatal mechanical complication of myocardial infarction (MI). This event occurs two to eight days after infarction and is more likely to occur in the anterior septum than in the posterior septum (60% vs. 40%) and often precipitates cardiogenic shock [1]. The differential diagnosis of postinfarction cardiogenic shock includes free ventricular wall rupture and the rupture of the papillary muscles. The incidence of postinfarct VSR had declined over the years due to early reperfusion capabilities. Postinfarction VSR is a surgical emergency, and the presence of cardiogenic shock is an indication for emergent intervention [2,3]. The best survival chances are reported when patients undergo early surgical repair.

## Case presentation

An 87-year-old Caucasian woman with a known history of hypertension and arthritis presented to the emergency department with non-exertional, epigastric, non-radiating, sharp chest pain for the past several days. The patient stated that her chest pain was relieved by sublingual nitroglycerine. The patient stated that she was scared to come to the emergency department, as she didn't want to catch coronavirus disease 2019 (COVID-19) from the hospital. The patient denied any shortness of breath or palpitations. Her physical examination was unremarkable.

**Figure 1:**
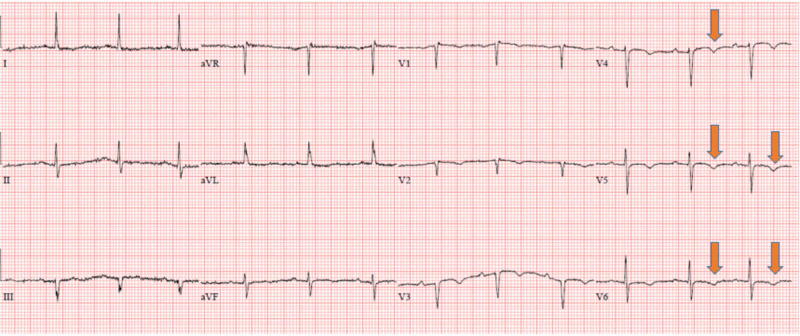
EKG showed normal sinus rhythm, normal axis, and intervals, but showed T-wave inversion in V3, V4, V5, and V6. EKG, electrocardiogram

**Figure 2:**
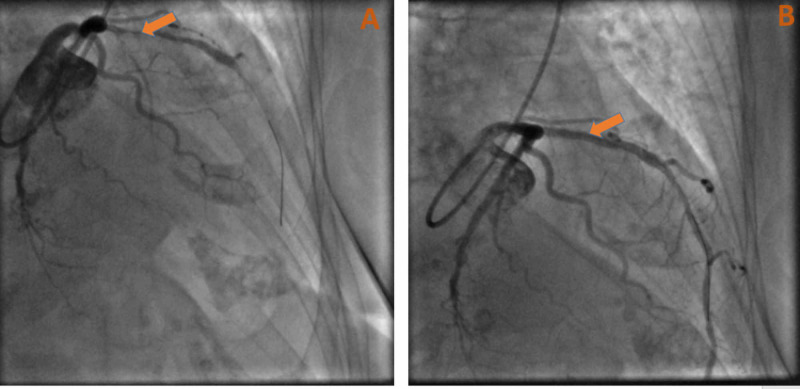
(A) Cardiac catheterization showed severe stenosis of the mid-LAD with the dominant circumflex artery. (B) Successful stenting of the mid-LAD with the return of blood flow distally. LAD, left anterior descending artery

Laboratory data on admission was notable for white blood cells (WBCs) of 15.19 cells/mcL (normal range: 4,500-11,000 cells/mcL), d-dimer of 3.08 mg/L (normal value < 0.50), initial troponin of 3.66 ng/mL (normal value < 0.05 ng/mL), and repeat troponin of 30 ng/mL. Electrocardiogram (EKG) showed normal sinus rhythm with T-wave inversions in the anterolateral leads (Figure 1). The patient was initiated on a heparin drip according to the acute coronary syndrome protocol and was titrated appropriately according to activated partial thromboplastin time (aPTT) results. A loading dose of aspirin was given with atorvastatin 40 mg and metoprolol tartrate 25 mg twice a day. The cardiology service was consulted, and subsequent cardiac catheterization showed significant stenosis of the left anterior descending artery (LAD) s/p placement of a drug-eluting stent (Figure 2). She was given dual antiplatelet therapy (aspirin 81 mg + clopidogrel 75 mg daily) post-percutaneous coronary intervention (PCI). Echocardiogram showed a reduced ejection fraction (EF) of 35-40%, with wall motion abnormalities in the LAD territory.

**Figure 3:**
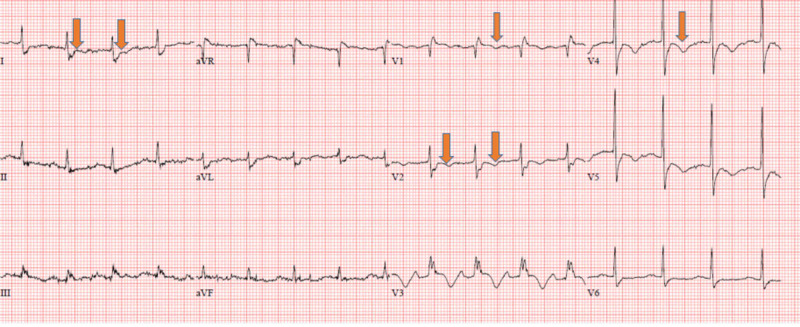
EKG showed sinus tachycardia with new ST-T wave abnormalities in the anteroseptal and lateral leads. EKG, electrocardiogram

**Figure 4:**
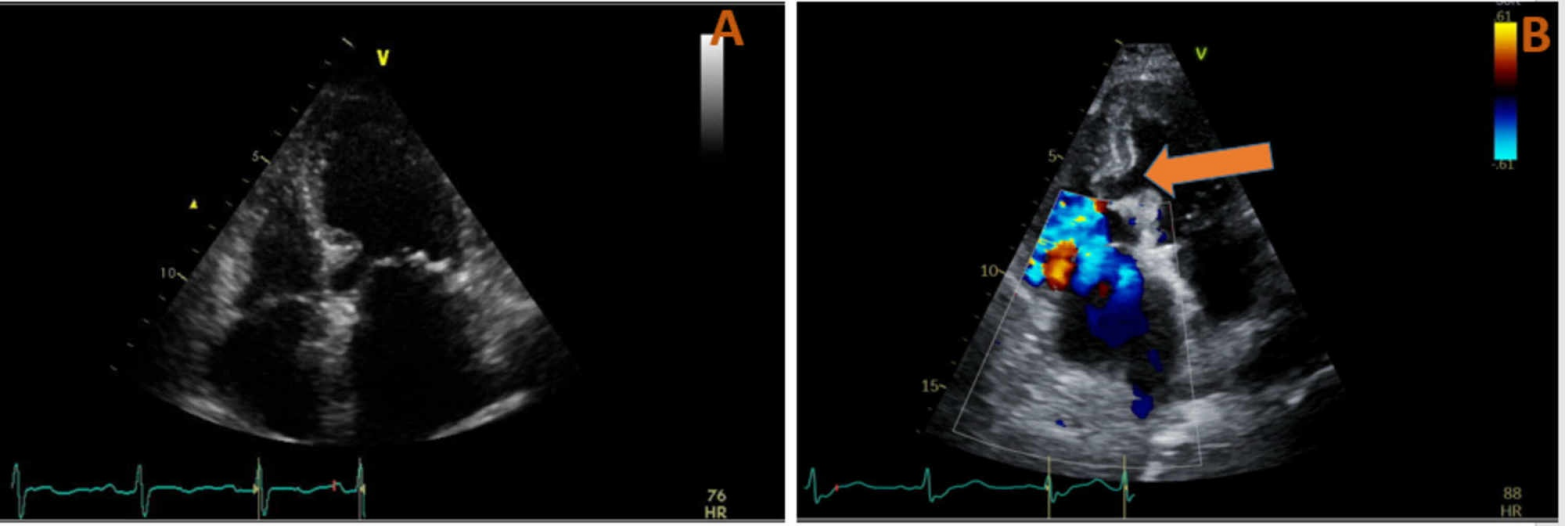
(A) Echocardiogram before cardiac catheterization showed reduced EF (35-40%) with an intact interventricular septum. (B) Doppler echocardiogram after a cardiac catheterization showed a significant reduction of EF (<20%) with new interventricular septum rupture, as shown by the white arrow. EF, ejection fraction

A few hours post-cardiac catheterization, the patient acutely decompensated and became less responsive. Her lab data showed acutely worsening leukocytosis up to 23.48 cells/mcL, sodium of 147 mmol/L (normal range: 135-145 mmol/L), creatinine of 2.40 mg/dL (normal range: 0.84-1.21 mg/dL), and troponin of 96.1 ng/mL. EKG showed new ST-T wave abnormalities in the anteroseptal and lateral leads (Figure 3). STAT echocardiogram showed a new membranous VSR (Figure 4 and Video 1).

**Video 1:** Doppler echocardiogram showing ventricular septum rupture, causing the significant left-to-right shunting, and precipitating cardiogenic shock.

The patient developed cardiogenic shock, requiring three vasopressors (epinephrine, dobutamine, and vasopressin). Unfortunately, she continued to deteriorate, and eventually triggered a code blue. The family was notified of her condition and they decided to change goals of care to comfort care measures. The patient expired a few minutes later.

## Discussion

VSR is an uncommon mechanical complication of MI. Studies show that post-MI septal wall perforations occur at a rate of approximately 1-2%. VSR usually occurs within the first 1-14 days, within the zone of necrotic myocardial tissue. Clinical studies report an average time of 2.6 days from MI to VSR. According to Serpytis et al., the risk factors for higher mortality from acute VSD include female sex, advanced age, arterial hypertension, anterior wall acute MI (AMI), absence of previous AMI, and late arrival at the hospital [4]. The age range of patients who sustain a postinfarction VSR is wide, ranging from 44 to 81 years [4].

The pathophysiology of postinfarction VSR is explained by near occlusion of the septal blood supply that usually comes from the LAD, the posterior descending branch of the right coronary artery, and the circumflex artery when it is dominant. Infarctions associated with a VSR are usually transmural and extensive. Around 60% of VSRs occur with infarction of the anterior wall and 40% with infarction of the posterior or inferior wall. Posterior VSR may be accompanied by mitral valve insufficiency secondary to papillary muscle infarction or dysfunction [5]. The postinfarct pathological changes that contribute to septal rupture involve coagulation necrosis of ischemic tissue with neutrophilic infiltration, eventually causing thinning and weakening of the septal myocardium, which usually happens within three to five days postinfarct. However, VSR occurring within 24 hours of presentation is more likely due to the dissection of an intramural hematoma or hemorrhage into the ischemic myocardium. These changes are explained by the physical shear stressors at the border of an infarct zone, combined with a hypercontractile, and remote myocardial segment. Clinically, it is usually seen with inferior infarction with the VSR noted in the inferior basal septum, abutting the hyperdynamic mid-septum that is perfused by the LAD [6,7]. Per Becker et al., the pathological features of cardiac free wall rupture, which is applicable to VSR, are categorized into three types. Type I rupture shows an abrupt, slit-like tear and occurs in acute infarcts at <24 hours. Type II rupture demonstrates the erosion of the infarcted myocardium and occurs in a sub-acute infarct within three to five days. Type III rupture is concomitantly associated with aneurysm formation, significant thinning of the septum, and subsequent rupture, which occurs in older infarcts over five days [8].

VSR results in biventricular failure due to left-to-right shunting, right ventricular (RV) volume, and pressure overload, thus increasing pulmonary venous return and secondary left-sided volume overload. The most obvious physical sign is a typically harsh, loud, and holosystolic murmur heard best at the lower left and usually right sternal borders, with occasionally widespread radiation. In some cases, the murmur is heard best at the apex and may be mistaken for acute mitral regurgitation. A thrill can be detected in up to 50% of patients. RV volume overload results in an accentuated pulmonic component of the second heart sound, left and/or right S3 gallop, and tricuspid regurgitation. On the other hand, the increased transmitral flow results in a mid-diastolic rumble. The hemodynamic stability depends on the size of infarction, the severity of left-to-right shunting, and RV dysfunction. In hemodynamically stable patients, the presence of a murmur or the findings of echocardiography may be the only tipoff to the early diagnosis. The physical signs of cardiogenic shock are profound hypotension, cold and clammy extremities, pulmonary edema, and oliguria. When the right ventricle is involved, the hepatic dysfunction and coagulopathy may occur [9].

There are no electrocardiographic characteristics of postinfarction VSR, but EKG can provide some clues. Persistent ST-segment elevation is common with a concomitant ventricular aneurysm. EKG can also help in predicting the anatomical location of the septal rupture. There are no electrocardiographic characteristics of postinfarction VSR, but EKG can provide some clues. Persistent ST-segment elevation is common with a ventricular aneurysm. EKG can help to predict the anatomic location of the septal rupture. The diagnosis can be confirmed with transthoracic echocardiography, which can identify ventricular septum defects in the 2D image and demonstrate the flow across the septum using color Doppler. In addition to that, it can identify evidence of RV dilation and pulmonary hypertension and exclude the other differentials of hemodynamic instability. When the patient has poor acoustic windows due to mechanical ventilation or body habitus, a transesophageal echocardiogram (TEE) should be considered. In patients undergoing coronary angiography, a left ventricular angiogram also can easily lead to the diagnosis by showing the shunting of contrast dye from the left ventricle to the right ventricle [10]. In uncommon cases where the suspicion is still high, and transthoracic echocardiogram or TEE may reveal no definitive diagnosis, confirmation of the diagnosis may require insertion of a pulmonary artery balloon catheter to document the left-to-right shunt. In hemodynamically stable patients, cardiac MRI is able to show the delineation of the infarcted tissue and also the ruptured septum. However, this is not a standard diagnostic tool [11].

Initial pharmacological therapy may be used as an attempt to stabilize the patient hemodynamically. Vasodilators can be used as it will improve the cardiac output by decreasing the afterload and the left-to-right shunt. On the other hand, inotropic agents should not be used alone as it will increase the cardiac output without changes in the ratio of pulmonary to systemic flow, thus increasing the left ventricular work and myocardial oxygen consumption. The severity of cardiogenic shock in some patients precludes vasodilator treatment and often mandates vasopressor support. Intra-aortic balloon pump (IABP) offers essential and temporarily hemodynamic support as a bridge to urgent intervention. IABP reduces the left ventricular afterload, thus increasing cardiac output and decreasing the left-to-right shunting. IABP also improves diastolic pressure, thus increasing the coronary blood flow and improving the myocardial oxygen supply [12,13].

Surgical intervention is the definitive treatment for postinfarction VSR. Arnaoutakis et al. reported on surgical outcomes in 2,876 VSR patients from the Society of Thoracic Surgeons National Database [14]. They concluded that early surgery is indicated to minimize the risk of mortality and morbidity. Thus, the diagnosis of VSR should prompt a heart team discussion of options. This discussion should take into account that, for some patients, surgery is futile as mortality approaches 100%. Very elderly patients and those with poor RV function often fall into this group. Elderly patients, female gender, shock, inferior infarction, pre-operative IABP use, pre-operative dialysis, mitral insufficiency, and timing of repair are risk factors for increased postoperative mortality [15,16].

Due to the unpredicted hemodynamic instability, early surgical repair should be considered in hemodynamically stable patients with preserved end-organ function and favorable anatomy. In patients undergoing surgery, coronary angiography should be performed if not already performed before the diagnosis of the mechanical complication [17,18]. If associated with severe coronary artery disease, an additional coronary artery bypass grafting for surgical closure of the rupture should be performed. Surgical repair of VSR is associated with relatively high mortality and suboptimal results, with a postoperative residual shunt in up to 20%. Given these poor results, the technique of percutaneous VSR device closure has been developed. Such a less invasive approach with a catheter-based intervention may offer improved survival or provide hemodynamic stabilization as a bridge to surgery. It might be used as an adjunctive therapy for residual post-surgical shunts [5,18].

## Conclusions

Although the incidence of postinfarct VSR has declined over the years due to early reperfusion capabilities, the delay in seeking medical care due to COVID-19 has the potential to reverse this trend. VSR is a life-threatening mechanical complication of MI leading to cardiogenic shock and subsequent death. Therefore, therapy must be initiated emergently. In addition to inotropic agents and vasopressors that could be used in patients with cardiogenic shock, IABP may be used as a temporary hemodynamic bridge for surgical repair. Surgical repair should be carried out on an emergency basis, even if the patient is hemodynamically stable. Patients who develop VSR with multiorgan failure may not be a candidate for surgery and should consider palliative care.
